# Evolution of T Cell Responses during Measles Virus Infection and RNA Clearance

**DOI:** 10.1038/s41598-017-10965-z

**Published:** 2017-09-13

**Authors:** Ashley N. Nelson, Nicole Putnam, Debra Hauer, Victoria K. Baxter, Robert J. Adams, Diane E. Griffin

**Affiliations:** 1 W. Harry Feinstone Department of Molecular Microbiology and Immunology, Johns Hopkins Bloomberg School of Public Health, Johns Hopkins University School of Medicine, Baltimore, MD 21205 USA; 2 Department of Molecular and Comparative Pathobiology, Johns Hopkins University School of Medicine, Baltimore, MD 21205 USA; 3 Present Address: Vanderbilt University School of Medicine, Nashville, TN 37232 USA; 4 Present Address: University of North Carolina School of Medicine, Chapel Hill, NC USA

## Abstract

Measles is an acute viral disease associated both with immune suppression and development of life-long immunity. Clearance of measles virus (MeV) involves rapid elimination of infectious virus during the rash followed by slow elimination of viral RNA. To characterize cellular immune responses during recovery, we analyzed the appearance, specificity and function of MeV-specific T cells for 6 months after respiratory infection of rhesus macaques with wild type MeV. IFN-γ and IL-17-producing cells specific for the hemagglutinin and nucleocapsid proteins appeared in circulation in multiple waves approximately 2-3, 8 and 18–24 weeks after infection. IFN-γ-secreting cells were most abundant early and IL-17-secreting cells late. Both CD4^+^ and CD8^+^ T cells were sources of IFN-γ and IL-17, and IL-17-producing cells expressed RORγt. Therefore, the cellular immune response evolves during MeV clearance to produce functionally distinct subsets of MeV-specific CD4^+^ and CD8^+^ T cells at different times after infection.

## Introduction

Measles is a highly contagious viral disease that remains an important cause of childhood morbidity and mortality^1^ with most deaths due to secondary infections^2, 3^. Measles virus (MeV), the causative agent of measles, is transmitted by the respiratory route and has an incubation period of 10–14 days. From the respiratory tract, MeV spreads to local lymphatic tissue and then to multiple organs including the skin. The prodrome of fever, cough and conjunctivitis is followed by a maculopapular rash associated with development of the adaptive immune response and T cell infiltration into sites of MeV-infected skin cells^4^. Although infectious MeV is cleared soon after the appearance of the rash, MeV RNA persists in peripheral blood mononuclear cells (PBMCs), urine and nasopharyngeal secretions of both naturally infected children^5, 6^ and experimentally infected rhesus macaques^7^ for several months.

The host adaptive immune response is necessary for control and clearance of virus^8, 9^ and both MeV-specific antibody and T cells contribute to gradual clearance of viral RNA from PBMCs^7^. Studies of both humans and monkeys suggest that CD8^+^ T cells are important for clearance of infectious virus during the rash. MeV-specific cytotoxic T lymphocytes appear in blood during natural infection^10^ and experimentally infected macaques depleted of CD8^+^ T lymphocytes have viremias that are higher and of longer duration than immunologically intact monkeys^11^.

Although less well studied, CD4^+^ T lymphocytes are likely to be essential contributors to a successful immune response to MeV and establishment of life long immunity. Naïve CD4^+^ T cells develop into functionally distinct subsets defined by the conditions required for differentiation, transcription factor expression and cytokines produced and important subtypes include Th1 cells producing interferon (IFN)-γ, Th2 cells producing IL-4, Th17 cells producing IL-17 and Treg cells producing IL-10^12^. Evaluation of cytokines in plasma of children with measles suggests that CD4^+^ T cells predominantly produce IFN-γ during the rash period followed by a later switch to IL-4, IL-10 and IL-13 secretion as antibody production matures suggesting early development of Th1 followed by Th2 and Treg CD4^+^ T cells^13–15^. The possible development of effector CD4^+^ T cells producing IL-17 during the response to MeV was suggested in a vaccine study, but Th17 responses have not been systematically evaluated^16^.

Because it is likely that the functional evolution of T cell subsets during the prolonged phase of MeV RNA clearance is important for eventual virus clearance, immune suppression and establishment of life-long protective immunity, we characterized cellular immune responses to MeV over a period of six months after infection of rhesus macaques with a wild type strain of MeV.

## Results

### Measles virus RNA persists in multiple tissues

**Figure 1:**
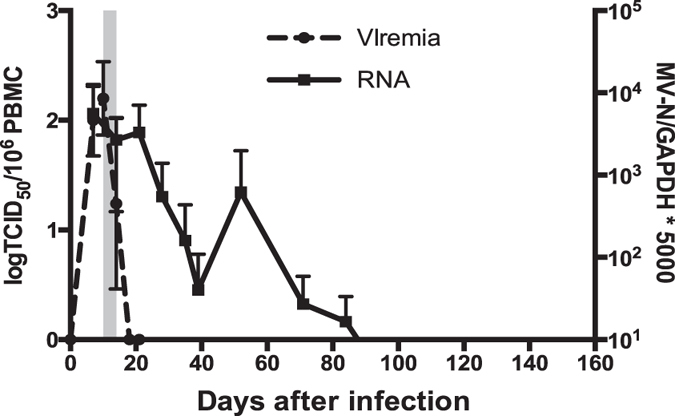
Measles viremia, rash and virus clearance. After intratracheal infection of rhesus macaques with the wild-type Bilthoven strain of MeV, viremia was measured by co-cultivation of serially diluted PBMCs on Vero/hSLAM cells. Data are displayed as the tissue culture infectious dose 50 (TCID_50_)/10^6^ PBMCs (n = 5). MeV N gene RNA in peripheral blood mononuclear cells was measured by RT-qPCR (n = 5). Gray shaded area indicates the period of the rash.

To document the time course of clearance of infectious virus and viral RNA in this cohort of 3-year old macaques, infectious virus in the blood was monitored by co-cultivation of PBMCs with Vero/hSLAM cells and viral RNA was quantified by RT-qPCR. All monkeys developed a viremia by day 7, a rash by day 10 and cleared infectious virus from PBMCs by day 18 (Fig. 1). MeV RNA was detected in respiratory secretions by 7 to 10 days after infection followed by continued shedding for 1–2 weeks (Table 1). MeV RNA in PBMCs gradually decreased after clearance of infectious virus and became undetectable 90 to 120 days after infection (Fig. 1). These data confirm that prolonged presence of viral RNA is characteristic of primary MeV infection^7^.

**Table 1:** Presence of MeV RNA in nasal secretions.

	14Y	17Y	31Y	46Y	50Y
0	−	−	−	−	−
7	−	+	−	−	+
10	+	+	+	+	+
14	+	+	+	+	+
18	−	+	+	−	+
21	−	+	−	−	+
39	−	−	−	−	−

### Changes in circulating leukocytes

**Figure 2:**
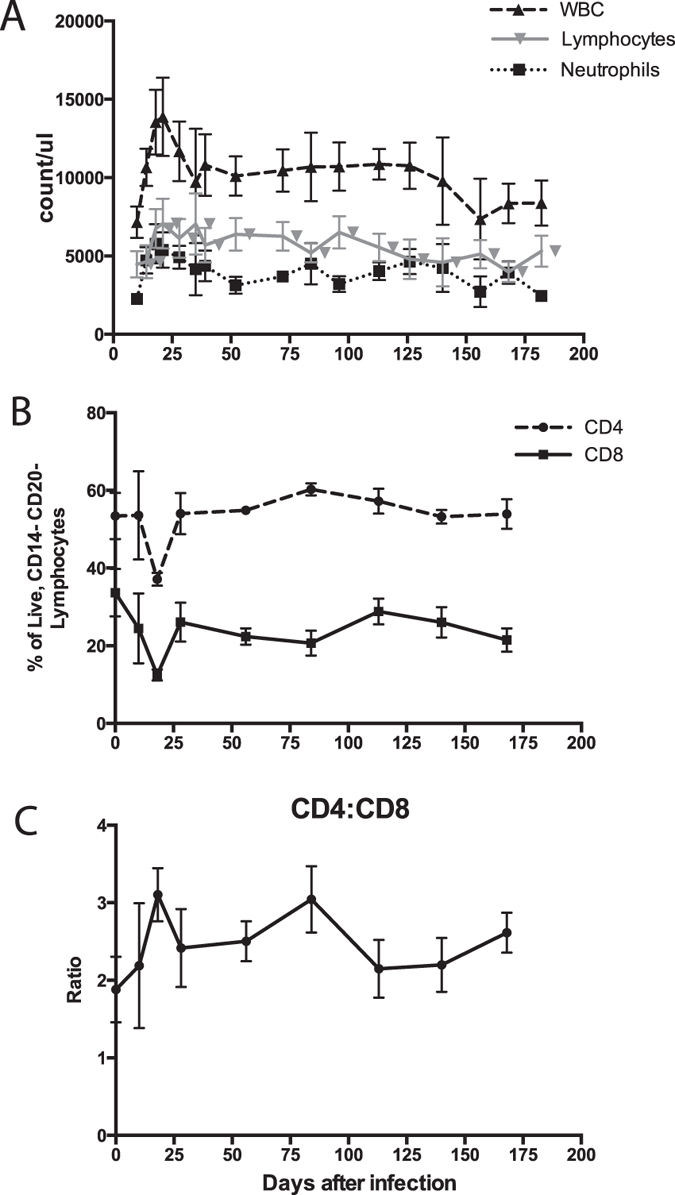
Changes in leukocyte counts after infection. Complete blood counts were used to measure the total numbers of circulating white blood cells (WBCs; dashed line), lymphocytes (gray line) and neutrophils (dotted line) (**A**) (n = 5). Normal ranges of cell counts for 3–4 year old rhesus macaques are: 7,700–13,000 WBCs/ml, 2,671–8,350 lymphocytes/ml, and 2,671–5,147 neutrophils/ml. Percentages of lymphocytes that were CD4^+^ and CD8^+^ T cells were determined by flow cytometry (**B**) and the CD4:CD8 ratio calculated (**C**) (n = 5). The normal CD4:CD8 ratio is 2.

Numbers of total white blood cells, lymphocytes, and neutrophils in circulation were depressed during the viremia (day 10), increased and then generally were within the normal range by four weeks after infection (Fig. 2A). Both CD8^+^ and CD4^+^ cells were decreased from day 10–18 and then returned to baseline (Fig. 2B). There was a transient increase in the CD4:CD8 ratio as the viremia was cleared and then stabilized in the normal range (Fig. 2C).

### ELISPOT analysis of IFNγ- and IL-17-secreting cells

**Figure 3:**
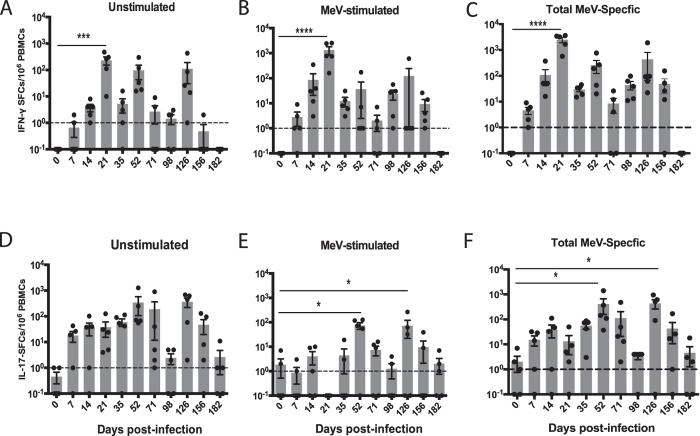
ELISPOT detection of IFN-γ and IL-17-secreting cells. PBMCs from MeV-infected macaques were cultured without *in vitro* stimulation (**A**,D) or were stimulated with H and N peptides (**C**) or MeV lysate (**F**) and cultured on plates coated with antibody to IFN-γ (**A**–**C**) or IL-17A (**D**–**F**) to determine numbers of spot-forming cells (SFCs) per 10^6^ PBMCs. To determine the numbers of cells secreting IFN-γ (**B**) or IL-17 (**E**) in response to *in vitro* MeV stimulation, numbers of SFCs in unstimulated wells (**A**,**D**) were subtracted from the numbers of SFCs in MeV H and N (**C**) or MeV lysate (**F**)-stimulated wells. *P < 0.05; ***P < 0.001; ****P < 0.0001. One-way ANOVA with repeated measures followed by Bonferroni multiple comparisons test (n = 5).

Cells secreting IFN-γ (Fig. 3A) and IL-17 (Fig. 3C) as a result of *in vivo* activation were present at multiple times after infection with peaks at days 14–21, 52 and 126. *In vitro* MeV antigen stimulation further increased the numbers of IFN-γ-secreting cells at day 21 after infection coincident with the clearance of infectious virus and decline in viral RNA levels (Figs 1 and 3B). In contrast, peak numbers of IL-17-secreting cells in response to *in vivo* activation, as well as *in vitro* stimulation, occurred later on days 52 and 126 as viral RNA was being cleared (Figs 1 and 3D).

### Both CD4^+^ and CD8^+^ T cells express IFN-γ during viral clearance and recovery

**Figure 4:**
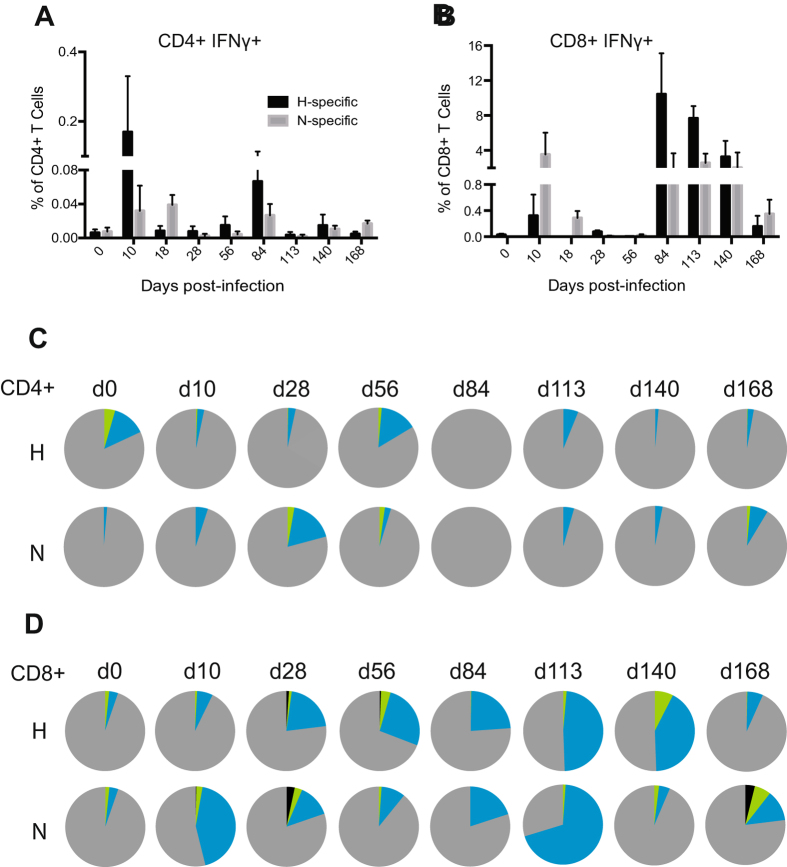
Assessment of the ability of MeV-specific T cells to produce effector cytokines over time by intracellular staining and multicolor flow cytometry. PBMCs were stimulated with pooled overlapping peptides from the MeV H or N proteins and IFN-γ-producing CD4^+^ cells (**A**) and CD8^+^ cells (**B**) were identified. The frequency of MeV specific IFN-γ-producing cells was determined by subtracting the spontaneous response. The polyfunctionality of the MeV-specific T cell response was assessed by determining the ability of CD3^+^ CD4^+^ cells to simultaneously express IFN-γ, TNFα, or IL-2 (**C**) and CD3^+^ CD8^+^ cells for their ability to simultaneously express IFN-γ, CD107a, TNFα, or IL2 (**D**). Pie charts show the functional composition of CD4^+^ and CD8^+^ T cells that simultaneously express one (gray), two (blue), three (green), or four (black) different functional markers at a given time point (**C**,**D**) (n = 4–5/time point).

To determine the cellular sources and MeV protein-specific responses of early and late IFN-γ production during MeV infection, PBMCs were stimulated with overlapping H or N peptides and analyzed by multi-parameter flow cytometry. CD4^+^ and CD8^+^ cells specific for both H and N proteins were sources of IFN-γ throughout the course of infection (Fig. 4). CD4^+^ T cells producing IFN-γ in response to MeV peptide stimulation were most abundant in circulation at 10 and 84 days after infection (Fig. 4A). An increase in IFN-γ^+^CD8^+^ T cells was observed in all macaques at 10 days after infection (Fig. 4B). A second wave of IFN-γ^+^CD8^+^ T cells appeared from 84–140 days after infection in four of the five macaques (Fig. 4B).

**Figure 5:**
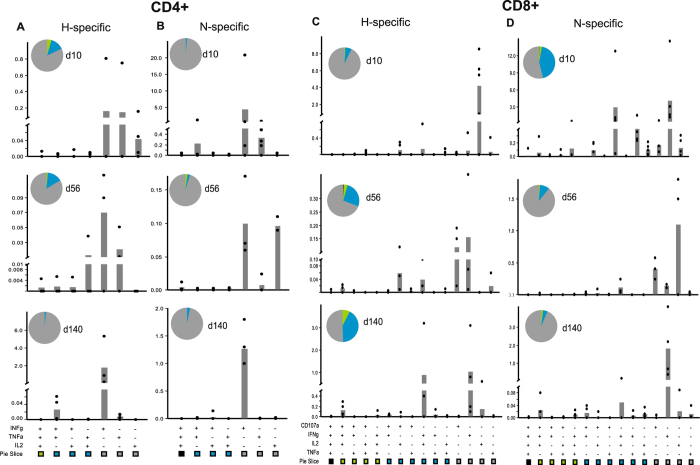
Functional analysis of MeV-specific T cells. PBMCs were stimulated with pooled H and N peptides. CD3^+^CD4^+^ cells were assessed for the ability to simultaneously express IFN-γ, TNFα, or IL-2 (**A** and **B**) and CD3^+^CD8^+^ cells for the ability to simultaneously express IFN-γ, CD107a, TNFα, or IL-2 (**C** and **D**). Subsets of cells expressing each functional marker were analyzed by Boolean gating. The frequency of each subset within CD4^+^ and CD8^+^ T cells is shown in the bar chart (n = 4–5/time point).

Several studies have suggested that the quality, as well as the magnitude of the T-cell response is important for control of virus infection^17–20^. An important measure of the functional quality of T cells is the ability to simultaneously produce multiple cytokines or polyfunctionality of these cells^17, 21^. H- and N-specific CD4^+^ and CD8^+^ T cell polyfunctionality was assessed by measuring simultaneous expression of IFN-γ, IL-2, TNF-α (CD4^+^ and CD8^+^) and CD107a (CD8^+^ only). A large fraction of H-specific CD4^+^ T cells were polyfunctional (producing more than one cytokine) at 56 dpi (Figs 4C and 5A), while N-specific CD4^+^ T cells were more polyfunctional at 28 dpi (Fig. 4C). In contrast, a large fraction of H- and N-specific CD8^+^ T cells were polyfunctional at multiple time points after infection (Fig. 4D). Overall, CD4^+^ T cells were polyfunctional primarily at earlier time points (Figs 4C and 5) while CD8^+^ T cells were polyfunctional at both early and late phases of recovery (Figs 4D and 5). Thus, MeV infection induces prolonged multifunctional virus-specific T cell responses likely to be important for controlling and clearing infection and perhaps for induction of long-term protective immunity.

### MeV-specific CD4+ and CD8+ T cells begin producing IL-17 after clearance of infectious virus

**Figure 6:**
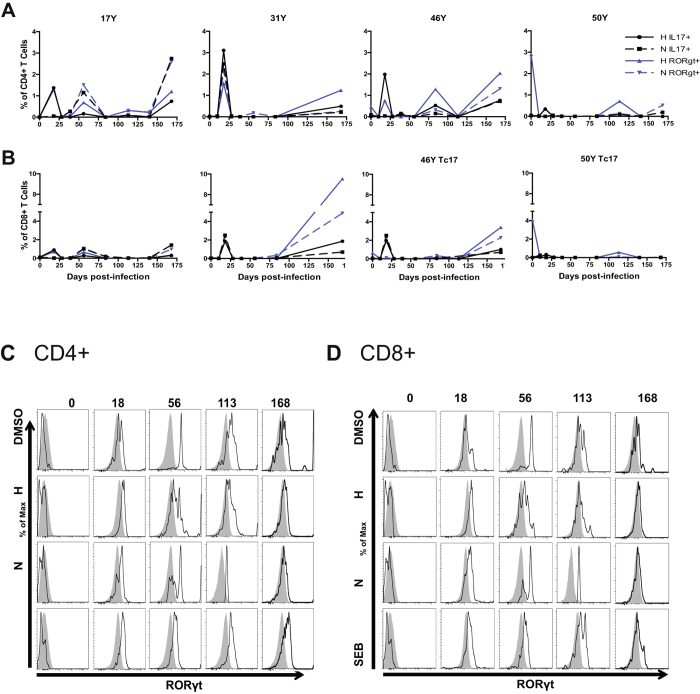
IL-17 and RORγt expression by MeV-specific T cells. PBMCs were stimulated with DMSO diluent, pooled overlapping peptides from the MeV H or N proteins or staphylococcal enterotoxin B (SEB) and IL-17^+^ and RORγt^+^ CD4^+^ (**A**) and CD8^+^ (**B**) cells were identified by intracellular cytokine staining and multicolor flow cytometry. CD4^+^IL-17^+^ (open) and CD4^+^IL-17^−^ (gray) populations (**C**), and in CD8^+^IL-17^+^ (open) and CD8^+^IL-17^−^ (gray) populations (**D**) are overlaid for comparison of RORγt expression.

To identify and further characterize the IL-17-producing cells induced during measles, surface and intracellular cytokine staining with multicolor flow cytometry was performed with complete data available for 4 of the 5 monkeys (Fig. 6). All monkeys developed MeV-specific Th17 (CD4^+^IL-17^+^) cells (Fig. 6A). H-specific Th17 cells increased from less than 0.01% (days 0 and 10) to 1.35–2.27% of the CD4^+^ T cell population at day 18, followed by intermittent increases above baseline throughout the follow-up period (Fig. 6A). N-specific Th17 cells were more variable. On average, Th17 H- and N- specific responses were highest on days 18 and 168 after infection.

MeV-specific Tc17 (CD8^+^IL-17^+^) cells were also induced after MeV infection with patterns of appearance in circulation similar to that of Th17 cells (Fig. 6A,B). All monkeys had an increase in H-specific Tc17 cells to 1.07–1.77% at day 18, whereas only monkey 31Y showed an increase in the frequency of N-specific Tc17 cells at day 18. Monkey 17Y had increases in both H- and N-specific Tc17 cells at day 56, while the other monkeys did not. All monkeys showed an increase in MeV-specific Tc17 cell frequencies at day 168 (Fig. 6B). In summary, MeV H and N-specific CD4^+^ and CD8^+^ T cells producing IL-17 were detectable on day 18 when infectious virus was cleared and then again when viral RNA was no longer detectable in PBMCs.

### MeV-specific T cell expression of RORγt

To better characterize the IL-17-producing cells, expression of the canonical IL-17 transcription factor RORγt^22^ was examined (Fig. 6A–D). The triphasic appearance of MeV-specific Th17 cells was also seen for cells expressing RORγt. All monkeys exhibited an increase in H-specific (1.09% ± 0.23) and N-specific (2.50% ± 1.99) RORγt-expressing CD4^+^ cells at day 18 (Fig. 6A). Frequencies of RORγt-expressing cells in response to H and N peptide stimulation remained low at day 28 through 39 followed by variable increases at day 56 or day 84. After day 113, the frequencies of MeV-specific RORγt-expressing CD4^+^ T cells showed a steady increase to approximately 1–2% at day 168 (Fig. 6A).

MeV-specific CD8^+^ T cells also expressed RORγt (Fig. 6B). Frequencies of H and N-specific CD8^+^RORγt^+^ T cells were increased at 18, 56 or 64, and 168 days after infection. All monkeys displayed an increase in H-specific RORγt-expressing CD8^+^ T cells at days 18 and 168 (Fig. 6B). MeV-specific CD8^+^ T cells expressing RORγt averaged approximately 4% of the total CD8^+^ T cells, with individual frequencies ranging from 0.5–10%.

In addition, we also characterized the simultaneous expression of IL-17 and RORγt. To better visualize the dynamics of co-expression, histograms of CD4^+^IL-17^+^ cells and CD4^+^IL-17^−^ cells (Fig. 6C) and CD8^+^IL-17^+^ and CD8^+^IL-17^−^ cells (Fig. 6D) were created and overlaid. Prior to infection, all populations had very low levels of RORγt expression, but by day 18, RORγt expression was detected (Fig. 6C,D). At days 56 and 113, IL-17-positive cells had higher levels of RORγt than IL-17-negative cells in both stimulated and non-stimulated populations. However, at day 168, the *ex vivo* MeV-stimulation does not increase RORγt expression in IL-17-positive relative to IL-17-negative cells.

## Discussion

Studies of measles in humans and macaques have suggested that T cell responses are important for recovery but the characteristics and time of appearance of MeV-specific T cells has received limited attention^11, 23^. In this study, we have monitored the evolution of T cell responses in MeV-infected rhesus macaques for a period of six months. Cells secreting IFN-γ and IL-17 *ex vivo* and responsive to additional *in vitro* MeV stimulation appeared in circulation in multiple waves approximately 2–3, 8 and 18–24 weeks after infection without a change in total lymphocyte counts. IFN-γ-secreting cells entered the circulation within the first 2 weeks and were most abundant 2–3 weeks after infection coincident with clearance of infectious virus. Virus-specific CD4^+^ (Th1) and CD8^+^ (Tc1) T cells were sources of IFN-γ early in infection, while CD8^+^ T cells were the predominant sources of IFN-γ later in infection. IL-17-secreting cells were most abundant later, were both CD4^+^ (Th17) and CD8^+^ (Tc17), expressed the transcription factor RORγt and showed specificity for H and N MeV proteins. These data show an ongoing evolution of the virus-specific cellular immune response during MeV clearance and suggest that IFN-γ-production may be important for early viral control while IL-17 production may be more important late during clearance of viral RNA. Prolonged multi-functional T cell responses may play an important role in maturation of the immune response to measles and establishment of life-long protective immunity.

Many IFN-γ-producing CD4^+^ and CD8^+^ cells were polyfunctional, secreting IL-2 and TNF-α as well as IFN-γ. These data are consistent with previous observations of increases in levels of TNFα mRNA in PBMCs and of IFN-γ, soluble IL-2 receptor, soluble CD8 and CD4 in plasma of children at the time of the measles rash^13, 14, 24–26^. IFN-γ has potent antiviral activities through induction of antiviral proteins and IFN-γ-secreting CD8^+^ T cells are also likely to have cytotoxic activity, both of which contribute to clearance of many virus infections^27^. *In vivo* depletion of CD8^+^ T cells has demonstrated their importance for control of measles virus^11^, simian immunodeficiency virus (SIV)^28^ and hepatitis B virus^29^ infections. Furthermore, CD4^+^ and CD8^+^ T cells can develop into memory T cells, which are more efficient producers of IFN-γ upon restimulation and provide protection from reinfection. The second increase in IFN-γ^+^CD8^+^ T cells in response to *ex vivo* stimulation 12–20 weeks after infection may reflect this population. Although more studies are needed to characterize these cells, it is likely that the T cell increase early after infection is important for the clearance of infectious virus while the later response may indicate renewed appearance of effector cells or development of a memory response.

The role(s) of IL-17-producing cells in virus infection are less clear, but they have been implicated in impaired virus clearance and immunopathology, as well as improved outcome^30^. Th17 cells are induced from naïve CD4^+^ T cells in the presence of IL-6 plus some combination of TGFβ, IL-21 and IL-1β a process that is inhibited by type I IFN^31^, and suppresses Foxp3 expression necessary for Treg development. The innate response that occurs after MeV infection may facilitate the development of IL-17-producing cells because IL-6 and IL-1β are increased early, but very little, if any, type I IFN is produced^15, 26, 32^.

The time course for generation of IL-17-producing T cells has not been carefully assessed for most infections; however, a few studies suggest that peak production is often late. For instance, in mice with keratitis due to herpes simplex virus infection, two waves of IL-17 mRNA (d2 and d21) are observed^33^. In children with respiratory syncytial virus-induced bronchiolitis, levels of IL-17 in nasopharyngeal secretions are higher in convalescence than during acute disease, in contrast to the other cytokines and chemokines measured^34^. Previous studies of macaques have shown increases in IL-17-producing cells at 10 and 35 days after MeV infection, but later times were not assessed^16^. In this study we have shown that IL-17-secreting cells were not only produced early in infection (day 18), but also late during the recovery process (day 168) and that both CD4^+^ and CD8^+^ T cells are sources of IL-17. It should also be noted that peak IL-17 secretion at 52 days after infection coincides with the reappearance of MeV RNA and is followed by a rapid decrease in viral RNA levels. These data further suggest the possibility that that IL-17 production may promote the clearance of viral RNA. While Th17 cells are polyfunctional, have multiple phenotypes and can play both detrimental and beneficial roles in disease pathogenesis^31, 35, 36^ their role in MeV infections is still unknown.

Virus-specific Th17 cells have been detected as part of the CD4^+^ T cell response to viral infections of mice^33, 37–41^, nonhuman primates^42–44^ and humans^45–47^. Expression of IL-17 by recombinant vaccinia virus leads to increased levels of virus in tissues^48^. Numbers of NKT cells producing IL-17 correlate with failure to control chronic SIV infection in macaques and higher levels of circulating Th17 cells correlate with higher viral loads and more severe liver disease in humans with chronic hepatitis B virus infection^49^. However, both improved clearance of influenza virus from the lung^50^ and no effect on clearance of herpes simplex virus from the cornea^51^ are also reported in response to IL-17-producing cells. The mechanism by which IL-17-producing cells may inhibit virus clearance is not clear.

Many innate immune cells including innate lymphoid cells, NK cells, and NKT cells can produce IFN-γ and/or IL-17. It is likely that these cells may contribute to the small burst of IFN-γ and IL-17 detected seven days after infection by ELISPOT assay. However, by 10 days after infection all macaques had developed rash, a hallmark for the onset of the adaptive immune response during measles infection^1^. Therefore, adaptive immune cells are likely to be the predominant producers of IFN-γ and IL-17 after the first week of infection.

Lastly, measles is associated with a transient period of immune suppression, which may last for several weeks to months after the acute stage of disease. Lymphopenia, a proposed contributor to immune suppression, is observed during the acute phase of infection and is associated with decreased numbers of T cells and B cells in circulation and lymphoid tissue^52^. We observed a transient decrease in T cell numbers at 10 days, which returned to baseline frequencies by 18 days with little variation despite a change in the method for identifying CD4^+^ T cells by flow cytometry.

These studies highlight the prolonged and complicated cellular immune responses generated by MeV infection. The immunologic processes driving the late development and repeated waves of IFN-γ and IL-17-producing cells and the effects of these responses on MeV RNA clearance are not known and merit further investigation. Although antibody responses were not addressed in this study, it is likely that immune-mediated clearance relies on the development of robust MeV-specific antibody responses as well as effective T cell responses^7^.

## Methods

### Ethics statement

For all procedures, monkeys were sedated with 10–15 mg/kg ketamine intramuscularly. All studies were performed in accordance with experimental protocols approved by the Johns Hopkins University Institutional Animal Care and Use Committee.

### Animals, infection and procedures

Five 3-year-old male measles-naïve rhesus macaques (*Macaca mulatta*) were obtained from the Johns Hopkins Primate Breeding Facility. The Bilthoven strain of wild-type MeV (genotype C2; gift of Albert Osterhaus, Erasmus University, Rotterdam) was grown in phytohemagglutinin-stimulated human cord blood cells and assayed by plaque formation on Vero/hSLAM cells^53^. Following baseline measurements, monkeys were infected intratracheally with 10^4^ plaque-forming units MeV in 1 ml PBS. Upon development of a rash, monkeys received either two daily doses of vitamin A (100,000 units, Vitamin Angels, Santa Barbara, CA; 14Y, 50Y) or placebo (17Y, 31Y, 46Y). No differences were observed between supplemented and non-supplemented macaques, so data have been pooled. Heparinized blood was collected from the femoral vein before infection and every 3–14 days after infection for six months.

### Sample processing and virus quantification

Beginning 10 days after infection, automated complete blood counts were performed by IDEXX Laboratories. PBMCs and plasma were isolated by whole blood gradient centrifugation on Lympholyte-Mammal (Cedarlane Labs). Infectious MeV in blood was measured by co-cultivating serially diluted fresh PBMCs with Vero/hSLAM cells for 5–6 days. Cytopathic effects in each well were assessed and the 50% tissue culture infectious dose (TCID_50_) calculated. Level of viremia is expressed as TCID_50_ per million PBMCs.

MeV RNA in PBMCs was measured by RT-qPCR as previously described^7, 54^. Briefly, RNA was isolated from 2 × 10^6^ PBMCs and the nucleocapsid (N) gene was amplified (Applied Biosystems Prism 7700) using a one-step RT-PCR kit with TaqMan primers and probe. Controls included GAPDH amplification and PBMC RNA from MeV-naïve monkeys. Copy number was determined using a standard curve constructed from 10^1^–10^8^ copies of RNA synthesized by *in vitro* transcription from a plasmid encoding the Edmonston N gene. The sensitivity of the assay was 50–100 copies. Data were normalized to the GAPDH control and expressed as [(copies of MeV N RNA)/(copies of GAPDH RNA)] × 5,000.

Nasal secretions were collected from both nostrils with sterile cotton swabs immersed in PBS. RNA isolated from the nasal cells (RNeasy Plus Micro Kit; Qiagen) was eluted in 30 μl of RNase-free water and 10 ng or 10 μl was used for RT-PCR. Primers MV41 (5′CATTACATCAGGATCCGG-3′) and MV42 (5′-GTATTGGTCCGCCTCATC-3′) were used to amplify a 350 base pair N gene sequence, and human β-actin RT-PCR primers (Agilent) were used as a control for RNA quality. PCR products were run on gels and read as positive or negative.

### ELISPOT assays

Enzyme-linked immunosorbent spot (ELISPOT) assays were used to identify PBMCs secreting IFN-γ and IL-17. Multiscreen HTS HA Opaque 96-well filtration plates (Millipore) were coated with mouse anti-human IFN-γ antibody (BD Biosciences, 2 μg/ml) or IL-17A antibody (eBioscience, 5 μg/ml) and blocked with RPMI/10% FBS. Cells were not stimulated or were stimulated with 1 μg/ml pooled hemagglutinin (H) or N overlapping peptides, 5.8 μg/ml MeV-infected Vero cell lysate (Advanced Biotechnologies) or 5 μg/ml concanavalin A. Freshly isolated PBMCs (10^5^) were added to wells stimulated with ConA, H or N peptides and 5 × 10^5^ PBMCs were added to non-stimulated and MeV lysate wells and incubated at 37 °C/5%CO_2_ for 40–42 h. Biotinylated anti-human IFN-γ (Mabtech, 7-B6-1; 1 μg/ml) or anti-human IL-17A (eBioscience, 64DEC17; 2 μg/ml) antibody was added for 2 h. Plates were developed with avidin-horseradish peroxidase (BD Biosciences; 1:2000) and diaminobenzidine substrate. Plates were read and analyzed using an ImmunoSpot plate reader and ImmunoSpot 5.0 software (C.T.L.). Data are presented as total spot-forming cells (SFCs)/10^6^ PBMCs. MeV-specific SFCs stimulated to secrete cytokines *ex vivo* were determined by subtracting the *in vivo* activated (no *in vitro* stimulation) SFCs from the total SFCs at each time point. All assays were done in duplicate.

### Flow cytometry

Multicolor flow cytometry with intracellular cytokine staining was used to identify CD4^+^ and CD8^+^ T cells expressing IFN-γ, IL-17, and RORγt. PBMCs were stimulated for 12 h using pooled overlapping H or N peptides (1 μg/ml), peptide diluent dimethyl sulfoxide (DMSO) or staphylococcal enterotoxin B. Except where noted, all reagents were from BD Biosciences or eBioscience. Mouse anti-human CD28 (CD28.2) and anti-human CD49d (9F10) were included with the peptides and DMSO. All stimulation mixes included GolgiStop and GolgiPlug.

Live/Dead Fixable Violet Dead Cell Stain Kit (Invitrogen) was used to eliminate dead cells from the analysis. Prior to surface staining, cells were incubated with human FcR block. All panels included anti-CD3 antibody (SP34-2). For days 0–18 a “dump gate” was used to eliminate cells labeled with anti-human CD14 (M5E2), anti-human CD20 (2H7) or anti-human CD8 (SK1) and CD4^+^ cells were defined as CD14^−^CD20^−^CD3^+^CD8^−^, because the anti-CD4 antibody initially used (RPA-T4) stained monkey CD4^+^ T cells poorly. From day 28 onward, anti-human CD4 (Biolegend, OKT4) was used and CD4^+^ T cells were defined as CD14^−^CD20^−^CD3^+^CD4^+^. CD8^+^ cells were defined as CD14^−^CD20^−^CD3^+^CD8^+^.

Intracellular staining was done following fixation and permeabilization of cells. For days 7–21 the Cytofix/Cytoperm Fixation and Permeabilization Kit and subsequently the Foxp3 Staining Buffer Set was used. Intracellular staining was done to detect the transcription factor RORγt (Q21-559) and the cytokines IFN-γ and IL-1-. T cell functionality was determined by gating on CD3^+^CD4^+^ and CD3^+^CD8^+^ cells stained for expression of IFN-γ, tumor necrosis factor alpha (TNFα), IL-2 or CD107a. Boolean gating was used to define all possible subsets. Percentages of cells that expressed one, two, three or four different functional markers were grouped and relative frequencies for each subset within CD3^+^CD4^+^ and CD3^+^CD8^+^ T cell populations were calculated. Samples were run on a BD FACS Canto II flow cytometer and analyzed using BD FACSDiva, FlowJo and SPICE (version 5.1; NIAID, NIH) software.

### Statistical analysis

The significance of differences in IFN-γ and IL-17-secreting cells was assessed by a one-way ANOVA with repeated measures followed by Bonferroni’s multiple comparisons test (GraphPad Prism version 7.00). Means for each time point were compared to the day 0 pre-infection levels. A p-value < 0.05 was considered significant.
